# Put your money where your mouth is: surveillance of antibiotic resistance within the commensal *Neisseria*

**DOI:** 10.1128/spectrum.00725-26

**Published:** 2026-06-15

**Authors:** Molly R. Regan, Caroline J. McDevitt, Leah R. Robinson, Souwaibat Issifou, Crista B. Wadsworth

**Affiliations:** 1 Thomas H. Gosnell School of Life Sciences, College of Science, Rochester Institute of Technology 6925, Rochester, New York, USA; University of Georgia College of Veterinary Medicine, Athens, Georgia, USA

**Keywords:** commensals, *Neisseria*, antibiotic resistance, microbiome, horizontal gene transfer

## Abstract

Commensal *Neisseria* species are major reservoirs of adaptive genetic variation, including antimicrobial resistance, for their pathogenic relatives, yet they remain poorly characterized. This gap limits our ability to anticipate resistance mechanisms that may ultimately emerge in *Neisseria gonorrhoeae* and *Neisseria meningitidis*. Here, we analyzed 166 novel commensal *Neisseria* isolates collected from 31 study participants and measured minimum inhibitory concentrations (MICs) for seven antimicrobials: azithromycin, cefixime, ceftriaxone, ciprofloxacin, doxycycline, penicillin, and gentamicin. Resistance, defined using the Clinical and Laboratory Standards Institute guidelines, was highly prevalent for azithromycin (76%) and doxycycline (52%), while no resistance to gentamicin was observed. High-level doxycycline resistance was always associated with the inheritance of *tetM*. Reduced susceptibility to azithromycin was linked to an MtrD K823E substitution, and reduced susceptibility to ciprofloxacin was associated with GyrA T91I (*Neisseria subflava*) or S91V (*Neisseria mucosa*). Across all antimicrobials, MICs varied widely, indicating the presence of additional modulating mutations. Finally, the genetic determinants underlying low-level doxycycline resistance and reduced penicillin susceptibility remain unresolved. Overall, here, we continue to build on the foundation of surveillance efforts in the commensal *Neisseria* and continue to flesh out what is known and unknown about this early warning system—or canary in the coal mine—for emerging resistance and clinically consequential evolution in pathogenic *Neisseria*.

**IMPORTANCE:**

Commensal *Neisseria* species constitute a vast and dynamic reservoir of genetic diversity that can be exchanged with pathogenic relatives, *Neisseria gonorrhoeae* and *Neisseria meningitidis*. However, these commensals remain substantially undercharacterized, limiting our ability to anticipate the evolutionary trajectories of antimicrobial resistance in clinically important species. By systematically analyzing commensal isolates and defining phenotypic resistance patterns alongside their genetic determinants, this study, and others like it, function as an early warning system for the emergence and spread of antimicrobial resistance. The high prevalence of azithromycin and doxycycline resistance, identification of specific mutations associated with reduced susceptibility, and evidence of additional unexplained contributors to minimum inhibitory concentration variation highlight both known and cryptic pathways of adaptation. These findings underscore the necessity of integrating commensal surveillance into resistance monitoring frameworks, improving our capacity to forecast clinically consequential evolution and to inform stewardship, diagnostics, and therapeutic development before resistance becomes entrenched in pathogenic *Neisseria*.

## INTRODUCTION

The rise of antibiotic resistance (AR) in *Neisseria gonorrhoeae* represents a critical and escalating threat to global public health. This pathogen, the causative agent of gonorrhea, has demonstrated a remarkable ability to develop resistance to nearly every class of antibiotics used against it, including sulfonamides, penicillin, macrolides, tetracyclines, fluoroquinolones, and, more recently, extended-spectrum cephalosporins (1–3). As evolving resistance continues to render first-line treatments increasingly ineffective, the risk of untreatable gonococcal infections looms larger, underscoring the urgent need for novel therapeutic strategies and more comprehensive surveillance systems. A key aspect of addressing this crisis lies in understanding the molecular and ecological mechanisms through which *N. gonorrhoeae* acquires antimicrobial resistance (AMR). While clinical surveillance of AMR has historically focused on pathogenic *Neisseria*, a growing body of evidence points to commensal *Neisseria*—harmless colonizers of the oral cavity—as potential and, until recently, largely overlooked reservoirs of resistance genes (4, 5).

Commensal *Neisseria* species colonize the human naso- and oropharynx. These species are universally carried by healthy human adults and make up the most abundant genera within *Proteobacteria* in oral and pharyngeal samples (approximately 10% of operational taxonomic units) (6, 7). Though species definitions across the *Neisseria* are in flux, the most commonly described human-associated species include those in seven distinct clusters (i) *Neisseria gonorrhoeae*; (ii) *Neisseria meningitidis*; (iii) *Neisseria lactamica*; (iv) the *Neisseria polysaccharea* cluster, which has been proposed to contain multiple species; (v) the *Neisseria subflava* group, which also contains *Neisseria flava* and *Neisseria flavescens*; (vi) the *Neisseria mucosa*, *Neisseria sicca*, and *Neisseria macacae* group*,* with a physiologically and genotypically distinct variant *Neisseria mucosa var. heidelbergensis*; and (vii) *Neisseria elongata* (atypical rod) (8–11). More recently, novel species have been proposed, including *Neisseria benediciae*, *Neisseria blantyrii*, *Neisseria bergeri*, *Neisseria basseii*, *Neisseria maigaei*, *Neisseria uirgultaei*, and *Neisseria viridiae* based on genomic data (8). However, as noted by Miari et al. (9) (9), there are 44 described *Neisseria* species (both human and animal associated) according to the List of Prokaryotic Names with Standing in Nomenclature, and 47 named by the National Center for Biotechnology Information (both databases accessed December 2025), highlighting the complexity and diversity of the genus both phenotypically and genotypically. Interestingly, *Neisseria* species have shown some evidence of localization to distinct ecological niches within the mouth, with *N. meningitidis* and *N. gonorrhoeae* localized to the throat, *N. subflava* favoring the tongue dorsum, *N. cinerea* preferring the keratinized gingiva and buccal mucosa, and other *Neisseria* mostly localized to the dental plaque (12, 13). However, despite this niche specialization and other barriers to gene exchange (e.g., DNA-uptake sequence divergence [14] and variation in DNA methylation [15]), DNA donation across species’ boundaries is rampant and is a major factor influencing evolution within the genus (4, 5, 16, 17).

Closely situated niches in the naso- and oropharynx allow for horizontal gene transfer across members of the genus. Genomic DNA flows relatively freely between *Neisseria* species as they constitutively express their competence systems and remain competent for transformation through all stages of growth. Type IV pili (T4P) promote transformation and are formed by pilin subunits (PilE), which pass through the outer membrane through the PilQ pore complex (18, 19). Surface-exposed ComP proteins recognize and bind *Neisseria*-specific DNA uptake sequences on extracellular DNA (20). When the pilus retracts, it draws ComP-bound DNA into the cell. Once in the periplasm, single-stranded DNA bound by ComE is imported through the inner membrane and into the cytoplasm through a pore formed by ComA (21, 22). Homologous recombination integrates DNA into the genome via RecA-mediated pathways (RecBCD and RecF) (22, 23). Additionally, the plasmid pConj, which has a high carriage rate across the *Neisseria*, contains all of the necessary machinery for conjugation (i.e., the *tra*-like genes encoding T4P and T4SS [24–26]) and thus facilitates the spread of both this plasmid and others (i.e., the β-lactamase-containing plasmid p*bla* and the cryptic plasmid pCryp [27, 28]) across the *Neisseria*. Through these mechanisms, commensal *Neisseria* have donated alleles encoding resistance to critical antibiotics, including: azithromycin, ciprofloxacin, penicillin, tetracycline, and extended-spectrum cephalosporins (4). Indeed, mosaic alleles in pathogenic species, including efflux pumps and promoter regions (*mtr*) (29, 30), penicillin-binding protein 2 alleles (*penA*) (31–34), and *gyrA* mutations (35), have all been traced back to commensal origins. In addition, commensal-to-pathogen transfer of pConj and *pbla* plasmids, known resistance determinants, has been documented (28).

Despite their central role in the spread of antimicrobial resistance, commensal *Neisseria* species remain significantly under-sampled in surveillance efforts. For example, of the 63,311 *Neisseria* genomes deposited to NCBI as of December 2025, 48,529 are *N. gonorrhoeae*, and 13,654 are *N. meningitidis*. Thus, only 2% of currently available genomes represent commensals, while 98% are of pathogenic sequences. Furthermore, existing studies are often limited in size, geographic diversity, and phenotypic characterization, leaving major gaps in our understanding of the *Neisseria* resistome and its potential to contribute to treatment failures in pathogenic species. Large-scale efforts to collect, identify, and phenotype commensal *Neisseria* isolates are therefore essential to inform surveillance strategies, guide therapeutic development, and preempt resistance outbreaks. Multiple papers have called for expanded *Neisseria* surveillance programs (i.e., such as the Gonococcal Isolate Surveillance Project from the Centers for Disease Control and Prevention (CDC), and the global Gonococcal Antimicrobial Surveillance Program [GASP] and its European version [Euro-GASP] from the WHO) to add commensals including Kenyon and colleagues (36); Goytia and Wadsworth (5); and Wadsworth, Goytia, and Shafer (4); however, currently, there are no national or international efforts to do so.

Here, we add to a growing number of studies collecting data on commensal resistance carriage (9, 37–42). In brief, we collect oral *Neisseria* isolates from human participants using non-invasive sampling, characterize them taxonomically using high-throughput sequencing, and profile their susceptibility to a panel of clinically relevant antibiotics. Our findings aim to expand the current understanding of commensal resistance carriage and to contribute valuable data toward surveillance efforts designed to predict and prevent the emergence of resistance in pathogenic *Neisseria* populations.

## MATERIALS AND METHODS

### Collection of novel *Neisseria* isolates from study participants

Study participants (≥18 years old) were recruited from students, staff, and faculty at RIT’s main campus (USA: Rochester, NY) from the summer of 2024 to the fall of 2025. There were no exclusion criteria other than age. Participants were provided written informed consent prior to participation in the study. After consent, participants were asked to complete a voluntary demographic questionnaire.

For bacterial collection, participants agitated the surfaces of their mouths (i.e., teeth, the roof of their mouth, and the insides of their cheeks) for 30 s with their tongue, after which they provided a sample of roughly 1 mL of saliva into a sterile tube. Samples were inoculated onto LBVT.SNR—a media developed for the isolation of *Neisseria* commensals (43)—and grown for 48 h at 30°C. For each participant, up to 10 colonies were picked and re-plated on gonococcal medium base (GCB) agar plates supplemented with 1% Kellogg’s solution (hereafter GCB-K plates), then incubated in the same conditions. Once sufficient growth of each colony was achieved, samples were stocked in TSB with 20% glycerol and stored at −80℃. The ability of LBVT.SNR to support the growth of commensal *Neisseria* was assessed using known species from the CDC and the Food and Drug Administration’s AR Isolate Bank. Strains were streaked onto LBVT.SNR plates and were grown for 24 h at 30°C, and the emergence of colonies or a lawn was coded as evidence of successful growth.

### Antimicrobial susceptibility testing

Frozen stocks of oral *Neisseria* isolates were revived by streaking onto GCB-K agar plates and incubating at 30°C for 24 h. Cells from overnight plates were suspended in TSB to a 0.5 McFarland standard and inoculated onto a GCB-K plate with an Etest strip. Following 18–24 h of incubation at 30°C, the minimum inhibitory concentrations (MICs) of each replicate were recorded. We defined resistance using the Clinical Laboratory Standards Institute (CLSI) breakpoints for *N. gonorrhoeae* (44). Doxycycline and gentamicin do not have published CLSI breakpoints for *N. gonorrhoeae*, so previously published interpretative breakpoints were used (45, 46). Though breakpoints for *N. meningitidis* are also published for azithromycin, ceftriaxone, ciprofloxacin, and penicillin (44) applying *N. gonorrhoeae* breakpoints provides a more conservative estimate of clinically relevant resistance in commensals, as *N. meningitidis* breakpoints are lower in all cases except for ciprofloxacin. Reported MICs are the mode of 2–3 independent tests and were agreed upon by two independent researchers. Antibiotics assessed include azithromycin, ceftriaxone, cefixime, ciprofloxacin, doxycycline, penicillin, and gentamicin (Table S1).

### Whole-genome sequencing

Cell lysis was performed by suspending cell growth from overnight plates in TE buffer (10 mM Tris [pH 8.0], 10 mM EDTA) with 0.5 mg/mL lysozyme and 3 mg/mL proteinase K (Sigma-Aldrich Corp., St. Louis, MO). DNA isolation utilized the PureLink Genomic DNA Mini Kit (Thermo Fisher Corp., Waltham, MA) with RNase A treatment to remove RNA. Isolated DNA was prepared for sequencing using the Nextera XT kit (Illumina Corp., San Diego, CA), uniquely dual-indexed, and pooled. The final pools were subsequently sequenced on the Illumina Novaseq or NextSeq platforms at the Rochester Institute of Technology Genomics Core using V3 600-cycle cartridges (2 × 300 bp) or 1,000/2,000 P2 XLEAP-SBS Reagent Kit (2 × 300 bp) cartridges, respectively. Read libraries were deposited to the NCBI’s Short Read Archive with accessions for each library reported in Table S1.

### Bioinformatics

Quality of each paired-end read library was assessed using FastQC v0.11.9 (47). Adapter sequences and poor-quality sequences were trimmed from read libraries using Trimmomatic v0.39, using a phred quality score <15 over a 4 bp sliding window as a cutoff (48). Reads <36 bp long, or those missing a mate, were also removed from subsequent analysis. Read libraries were assembled using SPAdes v.3.14 (49). Species identity for read libraries was defined using kraken2 (50) and subsequently confirmed using PubMLST’s *Neisseria* Typing tool (https://pubmlst.org/bigsdb?db=pubmlst_neisseria_seqdef&page=sequenceQuery) (51) (Table S1). Phylogenies were constructed using MLST loci (*abcZ*, *adk*, *aroE*, *fumC*, *gdh*, *pdhC*, and *pgm*) for all collected isolates in addition to 57 reference genomes (Table S2). In brief, loci were derived from each genome using blastn (52). Sequences were then concatenated, and the resultant multi-locus fasta file was aligned using MAFFT v.7.0 (53). A maximum likelihood tree was calculated using RAxML v.8.0 (54), and iTOL (55) was used to visualize and annotate the resultant trees. Blastn (52) was used to identify known resistance-associated mutations using loci from the *Neisseria gonorrhoeae* reference genome (NC002946) or all *Neisseria* alleles for a particular locus from the PubMLST database (1, 4). Orthologous sequence positions across taxa were aligned and rooted to the NC002946 reference, and the resulting alignments were visualized in Geneious Prime (56).

For commensal strains with evidence of polyphyletic species clustering, one isolate was chosen as a representative reference (*N. subflava*: P0006S004, P0009S001, P0014S002, P0019S002, P0033S006, P0036S002, and P0037S002; *N. mucosa*: P0005S001 and P0009S002), and reads from all isolates within a species cluster collected from the same sample participant were aligned back to the reference using Bowtie2 v.2.2.4, using the “end-to-end” and “very-sensitive” options (57). Pilon v.1.16 (58) was subsequently used to call variants from the reference sequence. All statistical analyses were conducted in R (59).

## RESULTS

### Participant demographics and minimum inhibitory concentration testing

Of the 31 study participants, 11 were female, 9 were male, and 11 participants did not report. Participant ages ranged from 18 to 36 (mean = 21.6). A total of 100% of study participants carried at least one commensal *Neisseria* isolate. Among the commensal isolates tested, resistance was the most prevalent to the antibiotic azithromycin, with 76% of isolates classified as resistant based on CLSI breakpoint guidelines for *N. gonorrhoeae* (MICs ranged from 0.38 to 256 µg/mL, Table 1). Resistance to doxycycline was the next most prevalent, with 52% of isolates found to be resistant (MICs: 0.023–256 µg/mL, Table 1). For β-lactams, 9% of isolates were resistant to penicillin (MIC range: 0.0125–4 µg/mL), 7% to cefixime (0.016–4 µg/mL), and 3.8% to ceftriaxone (0.008–1.5 µg/mL). Only 2.5% of isolates were resistant to ciprofloxacin (0.002–2 µg/mL), and no resistance to gentamicin was observed (0.75–8 µg/mL).

**TABLE 1:** Summary of antimicrobial resistance within the commensal *Neisseria* isolates collected for this study^*a*^

Antibiotic	*N*	MIC min (µg/mL)	MIC max (µg/mL)	MIC mean (µg/mL)	MIC median (µg/mL)	SD	No. resistant	Resistant (%)
Azithromycin	157	0.38	256	9.24	3	31.56	119	76
Cefixime	151	0.016	4	0.14	0.064	0.38	11	7
Doxycycline	157	0.023	256	14.75	1	39.6	81	52
Penicillin	154	0.125	4	0.99	1	0.63	14	9
Ceftriaxone	157	0.008	1.5	0.08	0.047	0.14	6	3.8
Ciprofloxacin	157	0.002	2	0.12	0.012	0.3	4	2.5
Gentamicin	155	0.75	8	2.2	2	0.88	0	0

^*a*^ AZI ≥ 2, PEN ≥ 2, CRO > 0.25, CFX > 0.25, CIP ≥ 1; DOX ≥ 1 and GEN ≥ 32 (Doxycycline and gentamicin have no established clinical breakpoints for *Neisseria*).

### Genomics data

**Fig 1:**
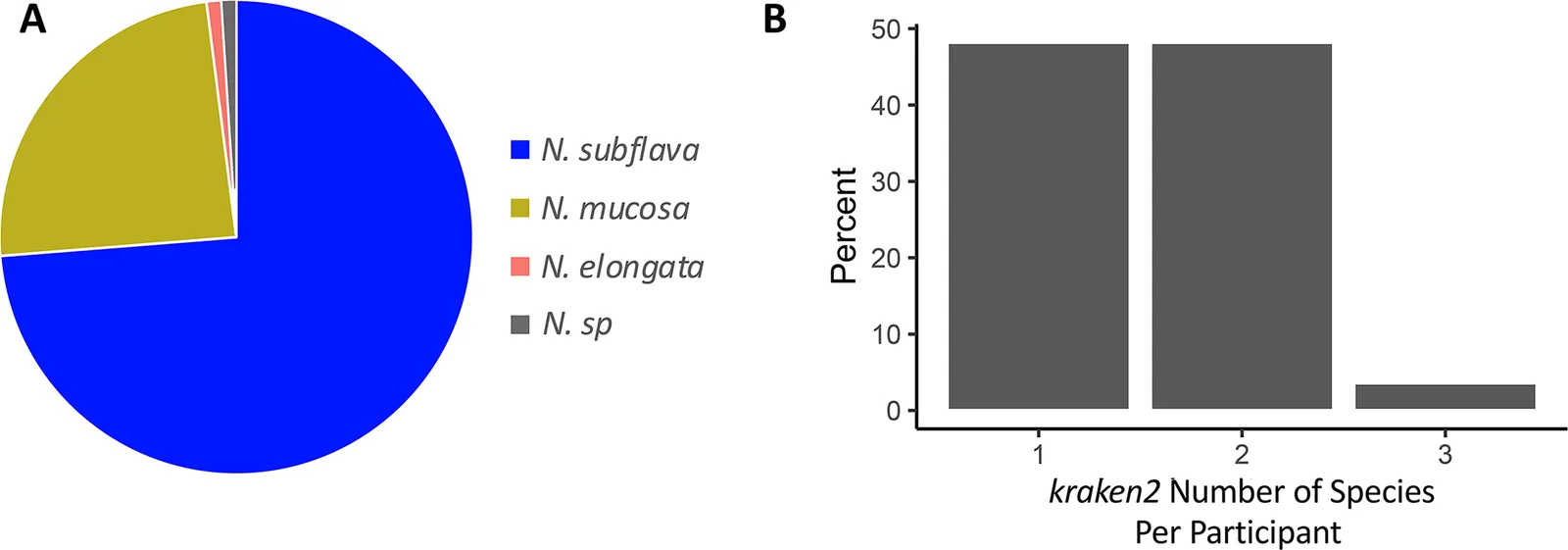
Neisserial species diversity for 166 isolates collected from Rochester, NY, between 2024 and 2025. (**A**) Kraken2 identified *N. subflava* to be the main species present among the study participants (121/165, 73%), followed by *N. mucosa* (40/165, 24%), *N. elongata* (2/165, 1%), and unclassified *Neisseria* species (2/165, 1%). (**B**) Excluding participants with only a single isolate collected (P0012 and P0023), 14 participants carried a single *Neisseria* species (*N. subflava*, 45%), while the remainder carried more than one. No participant carried more than three species.

**Fig 2:**
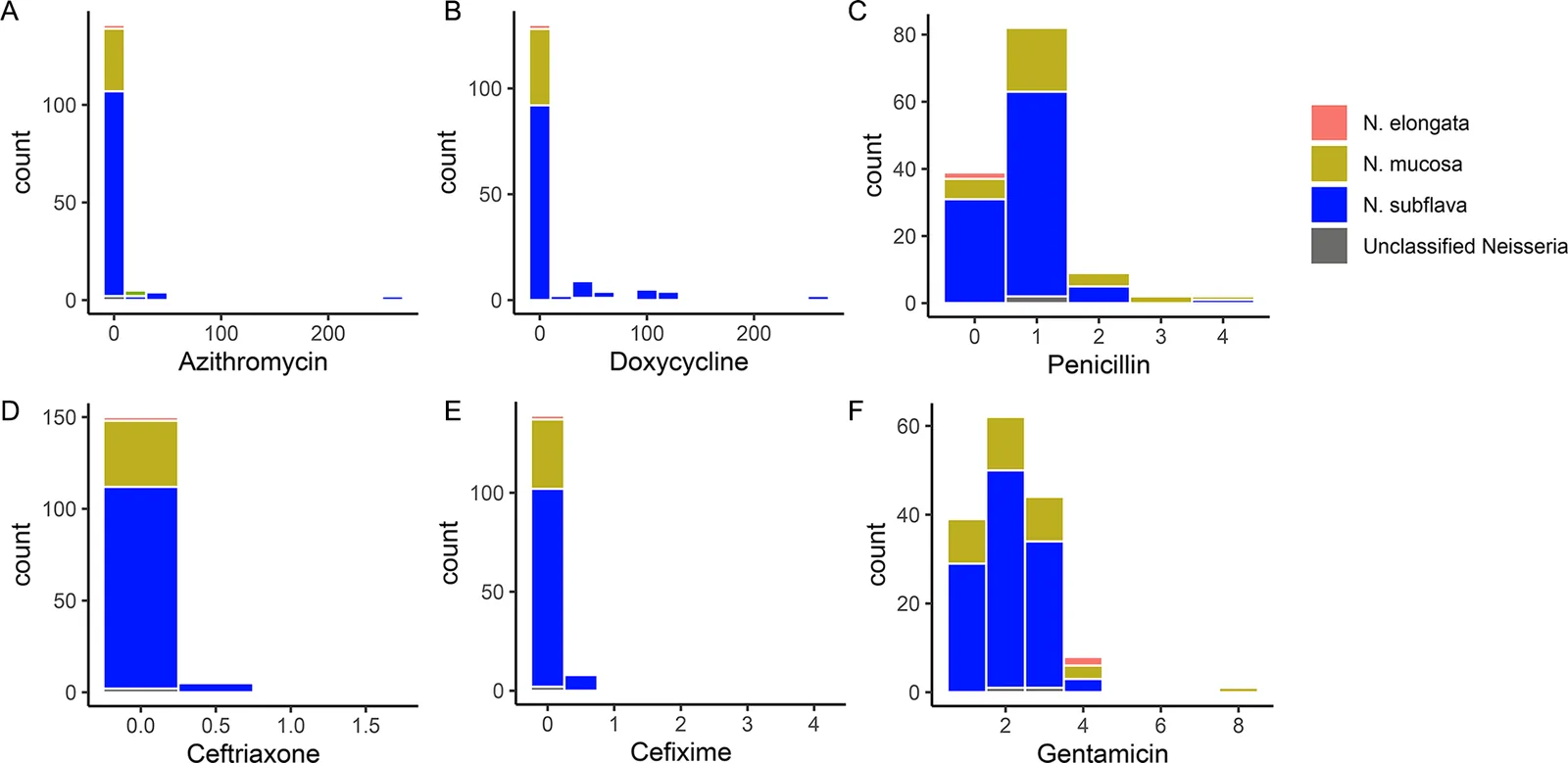
Minimum inhibitory concentration (MIC) distributions across *Neisseria* species for (**A**) azithromycin, (**B**) doxycycline, (**C**) penicillin, (**D**) ceftriaxone, (**E**) cefixime, and (**F**) gentamicin. *N. subflava* made up the highest percentage of resistant isolates for all antimicrobials: azithromycin 74% (89/119), ceftriaxone 100% (6/6), cefixime 100% (11/11), ciprofloxacin 75% (3/4), doxycycline 74% (60/81), and penicillin 50% (7/14).

A total of 166 samples were sequenced. The mean genome length was 2.44 Mb (range: 2.13–3.00 Mb). Kraken2-based species identification indicated that *N. subflava* was the predominant species (121/165, 73%; Fig. 1A). The second most common species was *N. mucosa* (40/165, 24%), followed by *N. elongata* (2/165, 1%) and unclassified *Neisseria* spp. (2/165, 1%). After excluding two participants with only a single isolate (P0012 and P0023), 14 participants carried a single *Neisseria* species (all *N. subflava*, 48.2%), 14 participants carried two species (*N. subflava* and *N. mucosa*, 48.2%), and 1 participant (P0008) carried three species, including *N. elongata* (Fig. 1B). Analysis of MIC distributions across species showed that *N. subflava* isolates had the highest proportion of resistant phenotypes across all antimicrobials: azithromycin 74% (89/119), ceftriaxone 100% (6/6), cefixime 100% (11/11), ciprofloxacin 75% (3/4), doxycycline 74% (60/81), and penicillin 50% (7/14; Fig. 2).

**Fig 3:**
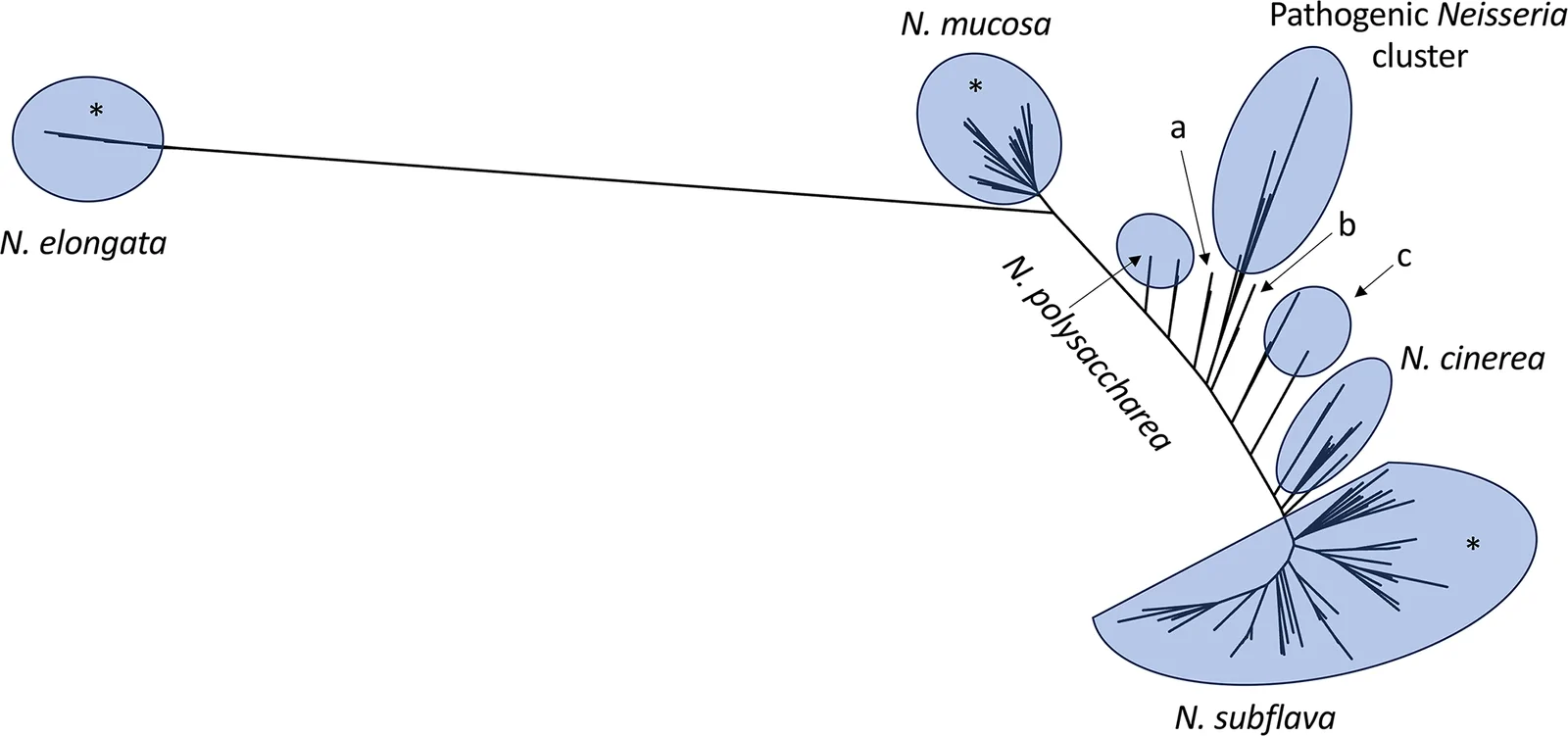
MLST gene-based maximum likelihood phylogeny for the 166 isolates collected within the study and 57 *Neisseria* reference isolates. Isolates recovered from participants on LBVT.SNR media included those clustering within the *N. elongata*, *N. mucosa*, and *N. subflava* species groups (indicated by *). We did not collect any isolates from the *N. cinerea* cluster, polyphyletic *N. polysaccharea* group, pathogenic *Neisseria* cluster (including *N. gonorrhoeae*, *N. lactamica*, *N. maigaei*, *N. meningitidis*, and *N. uirgultaei*), (a) *N. viridiae* group, (b) *N. benedictiae* and *N. blantyrii* group, or (c) *N. basseii* and *N. bergeri* polyphyletic cluster.

**Fig 4:**
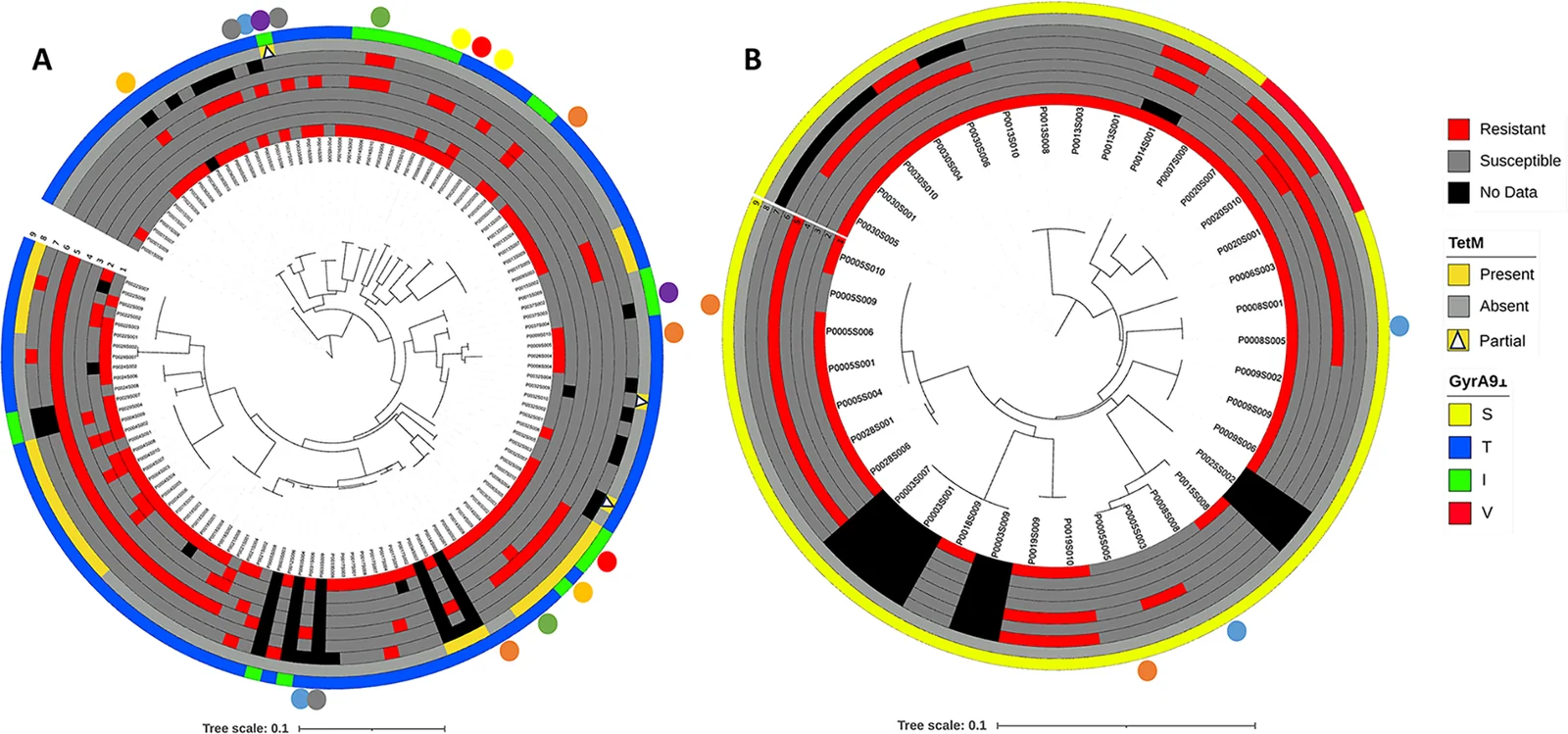
MLST maximum likelihood phylogenies by species for (**A**) *Neisseria subflava* and (**B**) *N. mucosa*. Annotation rings from the center indicate resistance (red) or susceptibility (gray) for (1) azithromycin, (2) cefixime, (3) ceftriaxone, (4) ciprofloxacin, (5) doxycycline, (6) gentamicin, and (7) penicillin. The eighth annotation ring indicates presence (yellow), partial sequence (yellow with white triangle), or absence (gray) of the *tetM* gene, followed by an annotation ring indicating the amino acids present at GyrA91. Colored circles on the outermost annotation rings indicate strains within participants that appear to be genetically distinct (i.e., clustering in multiple regions of the phylogeny).

Phylogenetic clustering confirmed kraken2 species’ identifications and clarified that the two unclassified *Neisseria* belonged to the *N. subflava* clade (Fig. 3). This analysis also confirmed that we did not collect any *Neisseria* from *N. cinerea*, *N. polysaccharea*, newly nominated *Neisseria* species (8), or the pathogenic *Neisseria* clade (*N. meningitidis*, *N. gonorrhoeae*, and closely related non-pathogenic *N. lactamica*; Fig. 3). As a note, *Neisseria* taxonomy changes substantially depending on the group, analysis, and data set. As per recent reports, we merged the *N. subflava* and *N. flavecens* species into a single group/cluster (9, 60) and merged the *N. mucosa*, *N. macacae*, and *N. sicca* species into a single cluster (9, 60). Reference genomes for these species were polyphyletic within groups, supporting this merging strategy. Within the *N. subflava* group, all 31 participants carried this species, with six showing evidence of multiple phylogenetically distinct strains (Fig. 4A). For the *N. mucosa* group, 15 participants carried this species, with two exhibiting multiple distinct strains (Fig. 4B). The two *N. elongata* isolates originated from a single participant and were closely related, although broader diversity could not be assessed due to limited sampling.

**Fig 5:**
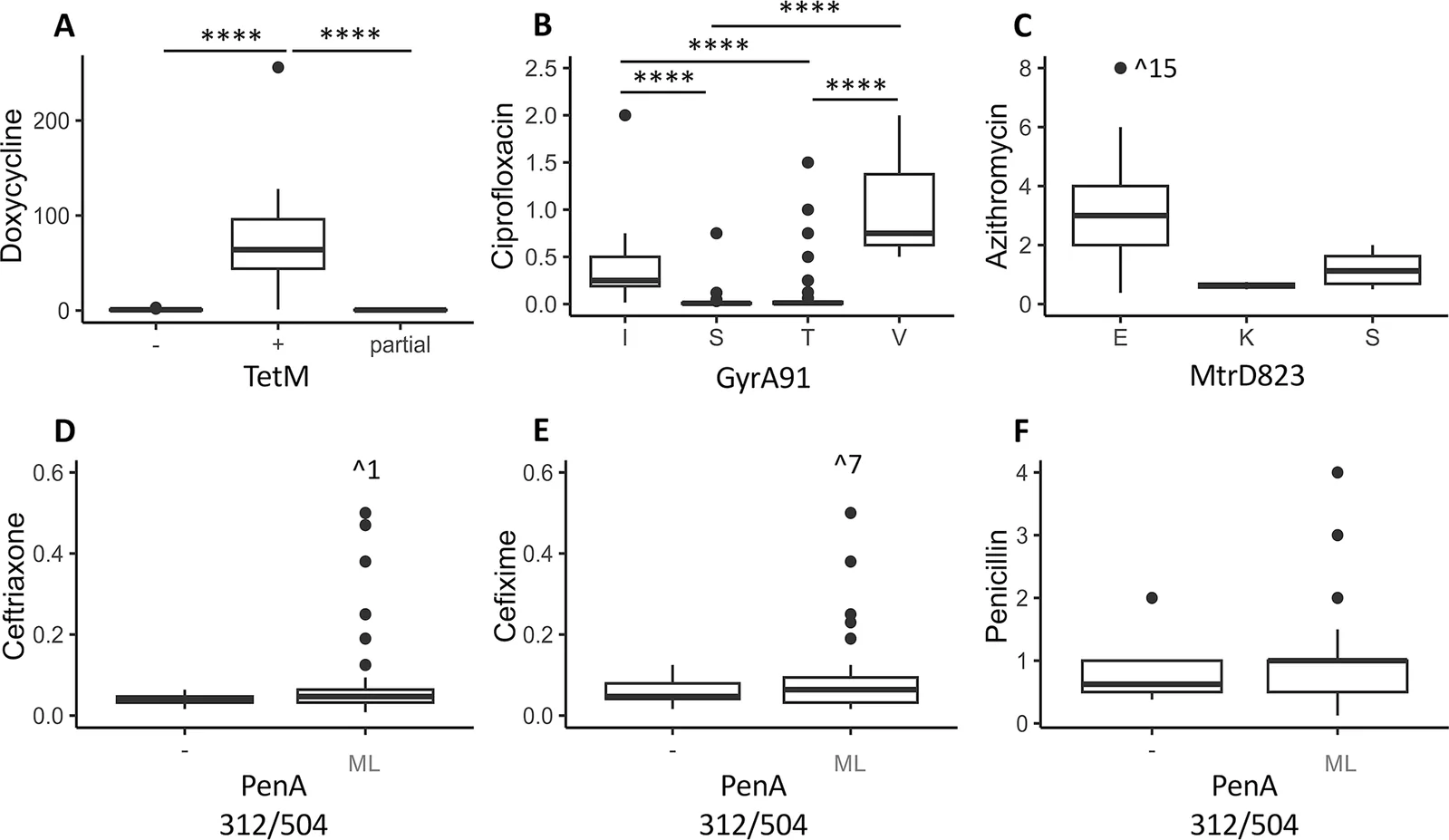
Associations between resistance mutations and MICs for isolates collected within the study. (**A**) High doxycycline MICs were significantly correlated with inheritance of the *tetM* gene. (**B**) Isoleucine and valine substitutions at GyrA position 91 resulted in significantly higher ciprofloxacin MICs. (**C**) Isolates inheriting MtrD823E had higher azithromycin MICs; however, this was not a significant difference. (**D–F**) PenA312M and 504L substitutions were always present in isolates with ceftriaxone and cefixime MICs above the breakpoint of >0.25 µg/mL. **** *P* < 0.0001.

BLAST was used to identify known resistance-associated mutations (4) using *N. gonorrhoeae* reference coordinates (NC_002946; Table S1). High-level doxycycline resistance was consistently associated with acquisition of the pConj plasmid encoding the ribosomal protection protein TetM, which was detected in 30 samples. Three isolates contained partial *tetM* sequences and had low MICs (<1 µg/mL), suggesting non-functional genes. Isolates with full-length *tetM* had significantly higher doxycycline MICs than those lacking the gene or carrying partial sequences (Tukey’s honest significant difference (HSD): *P* < 0.00001; Fig. 5A). MtrR A39T and G45D substitutions can also contribute (61); however, only wild-type alleles were observed in all *N. subflava* and *N. mucosa* samples. Both *N. elongata* isolates carried an A39N substitution but remained susceptible. Finally, elevated MICs can also be imparted by RpsJ V57M (62); however, all samples were monomorphic for wild-type alleles.

Ciprofloxacin resistance has been linked with the GyrA substitutions: S91F and D95A/N/G (63, 64). All isolates possessed *gyrA*. There was variability in our data set at position 91 with isolates harboring either S (*n* = 37), V (*n* = 3), T (*n* = 105), or I (*n* = 21) substitutions; however, all samples were wild-type at position 95. Isolates harboring a V or an I substitution at position 91 had significantly higher MICs than those with an S or a T (Tukey’s HSD: *P* < 0.00001 in all cases; Fig. 5B). Resistance-associated MtrR A39T, G45D, and Y105H (65) were not found. Finally, the resistance-associated GyrB mutation D429N was not found which imparts cross-resistance to zoliflodacin and gepotidacin, as well as other zoliflodacin-resistance-associated mutation S467N in the 128 isolates for which we were able to recover *gyrB* sequence (66, 67).

Multiple mutations have been associated with azithromycin resistance, including *23S rRNA* (C2611T and A2059G) (68), MtrR A39T and G45D (61), MtrD K823E (29, 30), RplD G68D/C and G70D (69), RplV (70), and RpmH (71) tandem duplications. No variation was observed at 23S positions 2611 or 2059, MtrR position 45, RplD positions 68 or 70, or in RplV or RpmH duplications. Two *N. elongata* isolates carried MtrR A39N but remained below resistance breakpoints. At MtrD only two isolates (both *N.elongata*) had the wild-type K amino acid at position 823; however, four isolates had MtrD K823S, and 160 had K823E. Though MICs for isolates with 823 S or E appeared elevated, they were not significantly different from wild type (Fig. 5C).

Gentamicin resistance has been associated with mutations in FusA A563V, G564D, and V651F (72); however, all were homogeneous for the wild-type allele in our data set.

For cephalosporins, we screened eight PenA substitutions (A311V, I312M, T316P, T483S, A501V, F504L, N512Y, and G545S) (73, 74). Of all of these positions, the only reduced susceptibility-associated mutations were found at positions 312 and 504, with all 158 isolates for which we had sequence data inheriting an M and an L substitution, respectively. We were unable to locate *penA* in eight assemblies. For isolates for which we had sequence data, inheritance of the M and L mutations was not consistently associated with elevated ceftriaxone, cefixime, or penicillin MICs, with a large range of MIC values for all drugs; however, the PenA 312M and 504L were always present in isolates with ceftriaxone and cefixime above the breakpoints of >0.25 µg/mL (Fig. 5D through F). RpoB R201H and RpoD E98K and Δ92–95 have also been associated with higher ceftriaxone MICs (75); however, these mutations were not found. Penicillin resistance can be caused by either chromosomal or plasmid-based mutations (76). We did not detect the β-lactamase-containing plasmid (p*bla*) in any of the samples reported in this study. Chromosomal mutations contributing to resistance include the aforementioned *penA* and *mtrR* mutations, as well as PonA L421P, which was not detected.

Carriage of resistance-associated mutations across species was distinct (Fig. 4). The *tetM* gene was detected exclusively in *N. subflava*. Carriage of tetM was associated with resistance in all cases except for three isolates, which carried *tetM* but had MICs of 0.25 µg/mL (P0032S009), 0.5 µg/mL (P0032S007), and 0.75 µg/mL (P0037S001). Only partial *tetM* sequences were recovered in these isolates, suggesting a non-functional gene. MICs for *N. subflava* isolates outside of these three isolates ranged from 1 to 256 µg/mL. For the extended-spectrum cephalosporins, only *N. subflava* had MICs above the breakpoint values. Finally, GyrA alleles were distinct across species, with *N. subflava* carrying GyrA 91 T and I and *N. mucosa* carrying S and V. Inheritance of I or V was associated with significantly higher MICs (Fig. 4B). *N. elongata* isolates were susceptible to all drugs investigated and only carried mutations associated with lower MIC values (Table S1).

## DISCUSSION

Commensal *Neisseria* species remain profoundly under-sampled and under-characterized relative to their pathogenic counterparts, despite mounting evidence that they play a critical role in the emergence and dissemination of antimicrobial resistance across the genus (4, 5). These organisms serve as a substantial and dynamic reservoir of resistance determinants, yet they are rarely included in routine surveillance or large-scale genomic studies (5). In this work, we directly address this gap by expanding the catalog of characterized *Neisseria* commensals. We collected and curated a set of novel commensal isolates and systematically surveyed them for antimicrobial resistance phenotypes and their associated genetic determinants. By pairing resistance profiling with genomic analysis, we aim to define both the breadth of resistance present in these species and the specific alleles that may be mobilized through horizontal gene transfer.

Reports of human colonization by commensals vary, ranging from 10.2% to 100% (9, 42, 77–79). However, as previously discussed by Miari et al. (9), this is likely due to the sampling strategy and/or media used. For example, studies highlighting a lower colonization rate used pharyngeal swabbing and modified Thayer Martin (TM) agar plates (77), which both will select against commensals due to the ecological niche specificity of these species (i.e., commensals are less frequently found in the pharyngeal niche compared to other oral sites [12, 13]) and the design of TM plates for isolation of pathogenic *Neisseria*. We add to a growing number of studies that support a 100% carriage rate for commensals (78). Species diversity, however, was low, with most isolates found to be *N. subflava* (123/165, 75%), followed by *N. mucosa* (40/165, 24%), then *N. elongata* (2/165, 1%), which we hypothesized was most likely due to exclusive usage of LBVT.SNR media and growth conditions at 30°C in a non-CO_2_ incubator, as at least the pathogenic *Neisseria* require nutrient-rich media and prefer higher temperatures and a 5% CO_2_ atmosphere (80). Commensals more closely related to the pathogenic *Neisseria* (Fig. 3) also failed to grow in the conditions used within this study. However, after testing the growth of known commensal strains on LBVT.SNR at 30°C, we observed evidence that the culture conditions and media supported growth of these other species (Table S3). Therefore, limited species diversity may be more likely to be due to the specific spit collection protocol used for this study. If *Neisseria* inhabit distinct niches within the oral cavity as some studies suggest (12, 13), it may be difficult to sample some species and sites (e.g., keratinized gingiva and/or gingival sulcus for *N. cinerea*) using a straightforward spit protocol; swabbing of these sites may, in fact, be more appropriate in these cases.

Investigation of interspecific diversity within each participant suggested that most carried predominantly *N. subflava* (48%) or a mix of *N. subflava* and *N. mucosa* (48%). *N. elongata* was rare and was only isolated from one participant, P0008; however, interestingly, that participant also had the highest interspecific diversity, carrying all three isolated species. Although characterized using different methodology (i.e., phenotypic rather than genotypic species identification), other studies also find the majority of people carry between 1 and 3 commensal *Neisseria* species using a similar collection protocol (9). Full analysis of species carriage diversity will likely have to use multiple collection protocols, due to the specific growth requirements of diverse *Neisseria* species and site specificity as discussed above.

**Fig 6:**
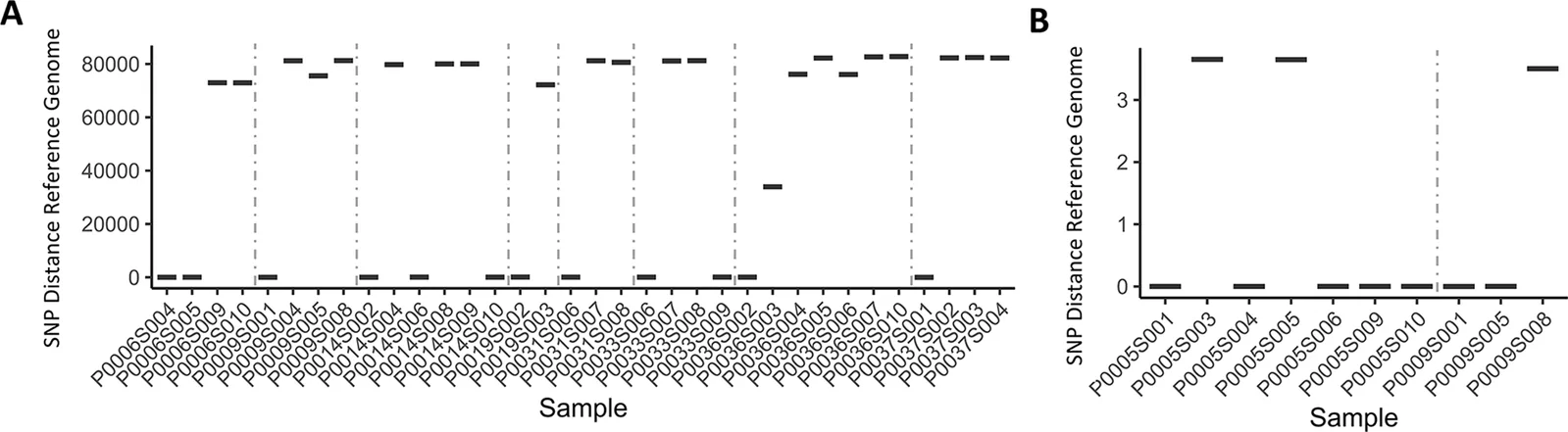
Within-host variation of *Neisseria* commensals indicates intraspecific divergence. (**A**) For *N. subflava*, eight participants carried at least two distinct strains. These strains carried on average 65,430 single-nucleotide polymorphisms (SNPs) compared to the participant-specific reference. (**B**) For *N. mucosa*, two participants carried at least two distinct strains separated by on average 37,670 SNPs. Overall, genomes ranged from 0.0009% to 3.65% divergent compared to reference sequences.

Within-host strain diversity has been assessed previously in *N. gonorrhoeae* (81, 82) and *N. meningiditis* (83, 84); however, these studies often focus on evolution across the course of colonization or infection, or on diversity between isolates colonizing different body sites in the case of *N. gonorrhoeae* (85, 86). Here, we assess intraspecific variation between isolates collected at the same time from the same location within individuals for *Neisseria* commensals. We find evidence of eight participants with at least two genetically distinct strains of *N. subflava* (P0006, P0009, P0014, P0019, P0031, P0033, P0036, and P0037), which supports prior reports of divergent *N. subflava* within a single host (87), and two participants with at least two genetically distinct strains of *N. mucosa* (P0005 and P0008), as indicated by polyphyletic clustering patterns (Fig. 4). For these participants, *N. subflava* isolates carried on average 65,430 single-nucleotide polymorphisms (SNPs) compared to a participant-specific reference (Fig. 6A); and *N. mucosa* strains were separated by an average of 37,670 SNPs (Fig. 6B). Overall, genomes ranged from 0.0009% to 3.65% divergent compared to reference sequences; with strains P0005S003 and P0005S005 on the high end of the range, differing by >100,000 SNPs compared to within-host reference (P0005S001). Generally, for bacteria, an average nucleotide identity of ≥95% is used to denote distinct species, so while our data suggest the possibility of distinct strains within-hosts, there is not enough divergence in this data set to suggest lineage splitting. Two possibilities exist for the observation of this intraspecific within-host diversity: (i) recent colonization of hosts with novel strains, with previous studies finding that commensals are shared between close contacts (88), or (ii) within-host oral colonization site divergence within a species, which has been demonstrated between species (12, 13), but not within a species at this point.

Surveillance of commensals may serve to inform on circulating resistance determinants, known or unknown, with the potential to disseminate into pathogenic *Neisseria* populations as has been observed for other resistance markers before (4, 5). In this data set, resistance was highly prevalent for azithromycin (76%) and doxycycline (52%), and while no resistance to gentamicin was observed, resistance was present to all other drugs investigated. As azithromycin and doxycycline are among the most frequently prescribed oral antibiotics prescribed in the United States (89), this may suggest that frequent use is selecting for elevated resistance carriage in commensal populations through bystander selection. Indeed, we have previously reported that doxycycline use is directly correlated with doxycycline resistance carriage and elevated doxycycline MICs in *Neisseria* (65), which we confirm in this expanded data set (Fig. S1). High-level doxycycline resistance >8 µg/mL was always associated with the inheritance of the *tetM* gene (Fig. 5A), while the mutations imparting low-level resistance are unclear, as RpsJ 57 mutations were not present. The prevalence of doxycycline resistance and the conjugative plasmid (pConj) harboring *tetM* is, of course, concerning due to the implementation of doxy-PEP—the prophylactic treatment with doxycycline within 72 h after sex for the treatment of sexually transmitted infections (90, 91). We can only anticipate that doxy-PEP selection will further spread the highly prevalent *tetM* and pConj in commensal populations, which has been predicted in mathematical models for *N. gonorrhoeae* (92). Furthermore, increased prevalence of pConj may also spread β-lactam resistance via transfer of p*bla*. Ultimately, we believe that doxy-PEP will likely increase the available pool of both of these plasmids and their resistance determinants for the pathogenic *Neisseria*.

Reduced susceptibility to ciprofloxacin in *N. gonorrhea* is frequently associated with GyrA S91F and D95A/N/G substitutions (63, 64). We did not observe any variability at position 95; however, there was variability in our data set at position 91 with isolates harboring either S (*n* = 37), V (*n* = 3), T (*n* = 105), or I (*n* = 21) substitutions. Reduced susceptibility was associated with GyrA T91I (*N. subflava*) or S91V (*N. mucosa*; Fig. 5B). In a previous report investigating alleles in the PubMLST database, we found that 3% of commensals harbor the resistance-associated GyrA 91I substitution (93), suggesting its availability in natural commensal populations. The presence of additional mutations was also noted. Reduced susceptibility to azithromycin was linked to an MtrD K823E substitution previously reported to contribute to resistance in *N. gonorrhoeae* isolates harboring mosaic *mtr* alleles (29, 30) (Fig. 5C). One gene that was absent in our analysis was PorB, as porins are highly divergent across species. For example, the *porB* allele between *N. gonorrhea* and *N. subflava* is over 75% divergent, making interpretation of alignments difficult; examination of this locus will be the subject of future work. Importantly, across all antimicrobials, MICs varied widely, likely indicating the presence of additional modulating mutations; and finally, the genetic determinants underlying low-level doxycycline resistance and reduced penicillin susceptibility remain unresolved.

In the absence of coordinated national or international strategies for commensal surveillance, the responsibility for documenting these species currently rests with individual laboratories. While fragmented, these efforts are essential. Together, they provide an early warning system, effectively a canary in the coal mine (5), potentially revealing resistance alleles circulating silently in nonpathogenic populations before they are detected in clinically significant pathogens. Ultimately, a more comprehensive understanding of commensal *Neisseria* (i.e., Kenyon et al.’s proposed “pan-*Neisseria*” approach [36]) will illuminate the full resistome accessible to the genus as a whole and identify genetic variants that may be poised for rapid transfer into pathogenic species. Such knowledge is crucial for anticipating future resistance trajectories and informing proactive antimicrobial stewardship and surveillance efforts.

## SUPPLEMENTAL MATERIAL

Fig. S1 spectrum.00725-26-s0001.pdf Doxycycline MICs in relation to antibiotic use.

Table S1 spectrum.00725-26-s0002.csv Data for *Neisseria* isolates collected within this study.

Table S2 spectrum.00725-26-s0003.xlsx NCBI accessions for reference *Neisseria* used within this study.

Table S3 spectrum.00725-26-s0004.xlsx Ability of AR-isolate bank commensals to grow on LBVT.SNR media at 30°C.

ASM does not own the copyrights to Supplemental Material that may be linked to, or accessed through, an article. The authors have granted ASM a non-exclusive, world-wide license to publish the Supplemental Material files. Please contact the corresponding author directly for reuse.
