# Supplementary material for: Put your money where your mouth is: surveillance of antibiotic resistance within the commensal Neisseria
Source: Microbiol Spectr. 2026 Jun 15;14(7):e00725-26. doi: 10.1128/spectrum.00725-26 (PMC13340140; doi:10.1128/spectrum.00725-26)
Supplement: Fig. S1 — Doxycycline MICs in relation to antibiotic use. [file spectrum.00725-26-s0001.pdf]

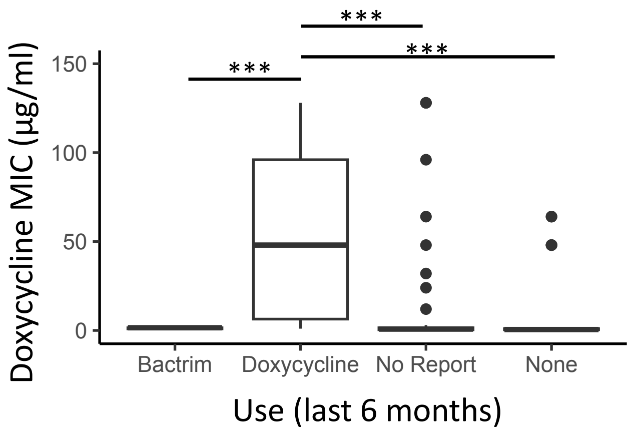

Supplementary Figure 1. Doxycycline use and correlation with elevated doxycycline MICs. Use was significantly associated with elevated MIC values.
